# Perioperative Vitamin C and E levels in Cardiac Surgery Patients and Their Clinical Significance

**DOI:** 10.3390/nu11092157

**Published:** 2019-09-09

**Authors:** Aileen Hill, Christina Borgs, Christina Fitzner, Christian Stoppe

**Affiliations:** 1 Department of Intensive Care Medicine, Medical Faculty RWTH Aachen University Hospital, D-52074 Aachen, Germany; 2 Department of Anesthesiology, Medical Faculty RWTH Aachen University Hospital, D-52074 Aachen, Germany; 3 3CARE—Cardiovascular Critical Care & Anesthesia Evaluation and Research, RWTH-Aachen University, D-52074 Aachen, Germany

**Keywords:** critical care, cardiac surgery, ascorbic acid, vitamin E, organ failure, prospective study, observational study

## Abstract

Background: Oxidative stress contributes to organ dysfunction after cardiac surgery and still represents a major problem. Antioxidants, such as vitamins C and E might be organ protective. Methods: The primary objective of this prospective observational study was the description to evaluate the perioperative vitamin C and E levels in 56 patients undergoing cardiac surgery with the use of cardiopulmonary bypass. The association of vitamin C with inflammatory reaction, oxidative stress, organ dysfunctions, and clinical outcomes were evaluated in an explorative approach. Results: Vitamin C levels decreased significantly from 6.5 (3.5–11.5) mg/L before surgery to 2.8 (2.0–3.9) mg/L 48 h after surgery (*p* < 0.0001). Fifty-six percent of patients had a suboptimal vitamin C status even before surgery. In protein-denaturized probes, significantly higher vitamin C concentrations were detected (*p* = 0.0008). Vitamin E levels decreased significantly from preoperative level 11.6 (9.5–13.2) mg/L to 7.1 (5.5–7.4) mg/L, (*p* = 0.0002) at the end of cardiopulmonary bypass, remained low during the first day on ICU and recovered to 8.2 (7.1–9.3) mg/L 48 h after surgery. No patient was vitamin E deficient before surgery. Analysis showed no statistically significant association of vitamin C with inflammation, oxidative stress or organ dysfunction levels in patients with previously suboptimal vitamin C status or patients with a perioperative decrease of ≥50% vitamin C after surgery. Patients with higher vitamin C levels had a shorter ICU stay than those who were vitamin C depleted, which was not statistically significant (72 versus 135 h, *p* = 0.1990). Conclusion: Vitamin C and E levels significantly declined intraoperatively and remained significantly reduced low for 2 days after cardiac surgery. The influence of reduced serum levels on the inflammatory reaction and clinical outcome of the patients remain unclear in this small observational study and need to be investigated further. Given vitamin C´s pleiotropic role in the human defense mechanisms, further trials are encouraged to evaluate the clinical significance of Vitamin C in cardiac surgery patients.

## 1. Introduction

Cardiac surgery is associated with oxidative stress and systemic inflammation, which both contribute to postoperative organ dysfunctions [1]. Stimuli leading to systemic inflammation reactions include the surgical trauma, foreign surface contact during cardiopulmonary bypass (CPB), ischemia-and-reperfusion injury (I/R)-injury, hemodilution and transfusion, as well as hemodynamic alterations [1,2,3]. Patients with extended surgical procedures and durations of CPB are exposed to a significantly higher inflammatory response with an increased risk of postoperative mortality and organ dysfunctions [4]. Attenuating oxidative stress and the resulting inflammatory response may therefore represent a promising protective strategy, especially in high-risk cardiac surgery patients to reduce the development of organ dysfunctions [2]. In the human biology, the release of free radicals after reperfusion is counteracted by the patient’s own natural antioxidant defense mechanisms [5]. Antioxidant nutrients, such as vitamins A, C, and E, selenium, glutamine, zinc, and copper are therefore consumed, and accordingly their serum levels decrease during and after cardiac surgery [6,7,8,9,10].

Current evidence suggests that the strong antioxidant and pleiotropic vitamin C can positively influence many organ systems, such as the neurologic, respiratory, renal, coagulation, and inflammatory and cardiocirculatory systems. The biochemical effects of vitamin C and the currently available preclinical and clinical evidence for the possible benefits of vitamin C administration in patients undergoing cardiac surgery was discussed in great detail elsewhere [11]. Briefly, while vitamin C’s strong antioxidant functions are derived from its ability to donate electrons and thereby acting as reductant, it is also required for reactions of more than 60 enzymes. For example, in the cardiovascular system, vitamin C is essential for the biosynthesis of noradrenaline, cortisol, vasopressin, and hormones that are crucial to maintain adequate vascular tone for organ perfusion [12]. Consequently, vitamin C levels may be closely linked to the vasopressor demand [13] and might be of particular relevance in patients with hemodynamic instability in the perioperative period of cardiac surgery. Additionally, low levels of vitamin C may predict the development of organ dysfunctions [14].

Vitamin E is a group of fat-soluble compounds, of which α-tocopherol is the most common and biologically active form to meet human vitamin E requirements [15,16], preventing lipid peroxidation during oxidative stress and thereby maintaining the integrity of polyunsaturated fatty acids in cell membranes [15]. In addition to its antioxidant effects, vitamin E seems to be involved in diverse signaling processes, as well as regulation of platelet aggregation. Furthermore, vitamin E activates protein kinase C, which is involved in a multitude of functions, such as the mediation of immune response, regulation of cell growth and gene transcription, modulation of membrane structure, learning, and memory [15,16,17,18]. The metabolism and regulation of plasma levels is complex and is thought to depend on the level of oxidative stress [15]. Vitamin E plasma levels can be regulated and restored by several substances including vitamin C [5,19,20,21]. Given the pleiotropic effects of vitamins C and E within the body’s anti-inflammatory and anti-oxidative defense mechanisms, this prospective observational study aimed to evaluate the perioperative time courses of vitamin C and E levels.

## 2. Methods

### 2.1. Patients

This prospective, observational study at University Hospital RWTH Aachen, Aachen/Germany enrolled patients undergoing cardiac surgery from September 2018 until May 2019. Written informed consent was obtained from all enrolled patients prior to surgery. All patients were scheduled for elective conventional open-heart surgery with the use of aortic cross-clamping, cardioplegic myocardial arrest, and CPB. Exclusion criteria were emergency operations, pregnancy, lack of informed consent, and age less than 18 years. The study was approved by the institutional review board (Ethics committee, RWTH Aachen University, Germany), registered at clinicaltrials.gov (NCT 02488876) and performed in adherence to the Declaration of Helsinki.

All patients received standard hospital meals during their entire hospital stay and were fasted for 6 h preoperatively. No patient received vitamin supplements during their hospital stay. All patients underwent catheterization, general anesthesia, and cardiac surgery with CPB according to local clinical standards. After surgery, all patients were transferred to a cardiac surgery intensive care unit (ICU) and were weaned from sedation and mechanical ventilation according to our department’s standards. Nutrition was provided in line with our feeding protocol, which is in agreement with current international guidelines on nutrition in the ICU.

### 2.2. Blood Sampling, Probe Handling, and Laboratory Measurements of the Vitamins

All blood samples for vitamin C and E measurements were drawn according to a standardized protocol prior to surgery, after opening of the aortic cross-clamp, at ICU-admission, and at 24 and 48 h after surgery.

The blood samples were collected in ethylenediaminetetraacetic acid (EDTA) tubes for vitamin C measurements and in serum tubes for vitamin E measurements. All blood probes were light-protected with tinfoil and immediately pushed into ice. The probes were centrifuged (3000 rpm for 10 min) as soon as possible (maximum allowed time until centrifugation: 60 min). The supernatant was transferred into an Eppendorf-tube and the probes were frozen at −80 °C under light protection. A planned interim analysis including all patients enrolled in 2018 revealed very low vitamin C levels. The method was afterwards modified based on the literature search as follows: protein was removed in the obtained serum after the first centrifugation using a detergent for protein denaturation (Chromsystems Instruments & Chemicals GmbH, Munich, Germany) in a 1:1 ratio. This step is necessary to precipitate protein, to stabilize the ascorbate, and to remove interfering substances, and uses either an acid or an alcohol. This procedure is often combined with the use of a metal chelator such as EDTA to prevent accelerated ex-vivo ascorbate oxidation by divalent metal cations [17,18]. Probes were well-mixed and centrifuged again (3000 rpm for 5 min) before storage under the above-described conditions. According to our standardized protocol, the maximum allowed time from probe drawing to storage in the freezer was 120 min to prevent degradation of the vitamins [22].

All probes were sent frozen and light-protected to an external laboratory (Labor Mönchengladbach MVZ Dr. Stein + Kollegen GbR, Mönchengladbach, Germany) for the determination of vitamin levels through high performance liquid chromatography. In current international and national recommendations, a fasting plasma vitamin C concentration below 50 µmol/L (equaling 8.81 mg/L) is an indicator of a suboptimal status with risk of insufficiency, while clinical symptoms such as fatigue might occur below 20 µmol/L [23,24,25]. The commonly used definition for vitamin C deficiency is a plasma vitamin C concentration below 11 μmol/L and a hypovitaminosis C is described below 23 μmol/L [26]. Reference values of plasma/serum α-tocopherol concentrations below 12 µmol/L (equaling 5.17 mg/L) may be indicative of α-tocopherol deficiency [21].

### 2.3. Measurement of Oxidative Stress and Inflammation

Blood samples were drawn in serum tubes and treated (centrifuged and frozen) as described above except that the probes were neither immediately light-protected nor pushed into ice. Oxidative stress was assessed using the oxidation–reduction potential (ORP) and antioxidant capacity (AC), measured with the RedoxSYS Diagnostic SystemTM (Aytu BioScience, Inc.,Englewood, CO, USA). A low ORP being a sum of all oxidants and reductants in the blood indicates low oxidative stress, while a high AC is a measure of good antioxidant defense.

Interleukin 6 (IL6) and interleukin 10 (IL10) were determined with commercially available ELISA assays (R&D Systems, Wiesbaden-Nordenstadt, Germany) according to the manufacturer’s instructions.

### 2.4. Data Collection

All other laboratory and clinical outcome parameters were collected as part of the clinical routine from chart review.

The inflammatory reaction was characterized through the measurement of the inflammatory mediators C-reactive protein (CRP), procalcitonin (PCT) as well as white blood count (WBC). Their measurement was performed at indication of the attending medical staff as part of the clinical routine.

Organ dysfunctions were assessed using the Sequential Organ Failure Assessment (SOFA) Score according to the original description [27]. SOFA score and incidences of the individual organ dysfunctions: delirium, stroke, requirement for catecholamines and fluids, atrial fibrillation, duration of mechanical ventilation, acute respiratory distress syndrome, pneumonia, acute kidney injury, and postoperative dialysis and infection were recorded as part of clinical routine in our electronic hospital database during the patient´s ICU stay. Clinically relevant outcome parameters, such as mortality, length-of hospital and ICU-stay, and duration of mechanical ventilation, were recorded and evaluated in an explorative approach.

### 2.5. Statistical Evaluation

We compared patients, with a preoperative suboptimal vitamin C status to patients with normal vitamin C levels (cutoff: 9 mg/L), as well as patients who had a perioperative loss of vitamin C of ≥50% to those patients who did not lose vitamin C.

Continuous variables were described by median and interquartile range (IQR) and categorical variables by counts and percentages. Group differences were tested by the Mann–Whitney U-test for continuous variables and the Fisher exact test for the categorical variables. Before–after comparisons were analyzed by paired Wilcoxon test. Due to the explorative study design, two-sided tests were used and *p* < 0.05 were considered statistically significant test results. The analysis was performed using SAS version 9.4 (SAS Institute Inc., Cary, NC, USA).

## 3. Results

### 3.1. Patient Characteristics

**Figure 1:**
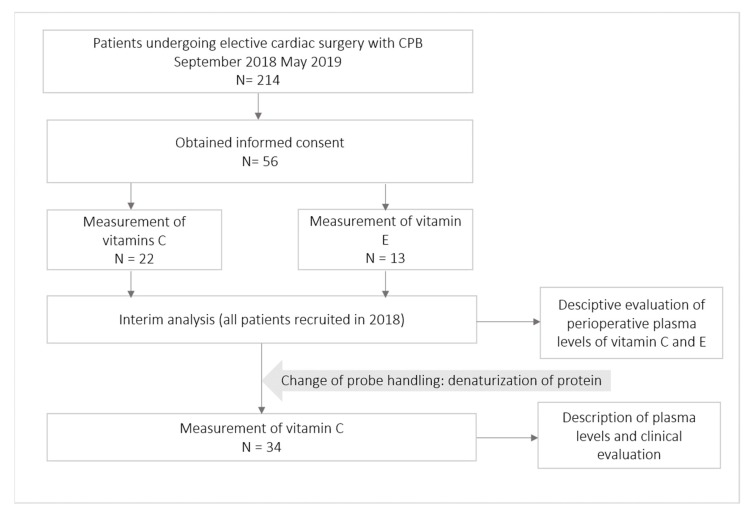
Patient flowchart. N, number of patients.

Fifty-six patients were consecutively enrolled in this study (Figure 1). In a scheduled interim analysis containing all patients recruited in 2018, very low vitamin C levels were detected, leading to a change of protocol, as described in Section ‘Blood Sampling, Probe Handling and Laboratory Measurements of the Vitamins’. Only those 34 patients in whose probes the protein was denaturized were considered for clinical and laboratory explorative analyses. However, the denaturation of proteins also impeded the measurement of vitamin E in these probes, due to which the analysis of clinical outcomes was not performed.

Patient characteristics are described in Table 1. All included patients reflect a representative cohort of cardiac surgery patients with typically preexisting disease and did not differ between patients with or without preoperative vitamin C deficiency.

### 3.2. Perioperative Course of Vitamins C and E

**Figure 2:**
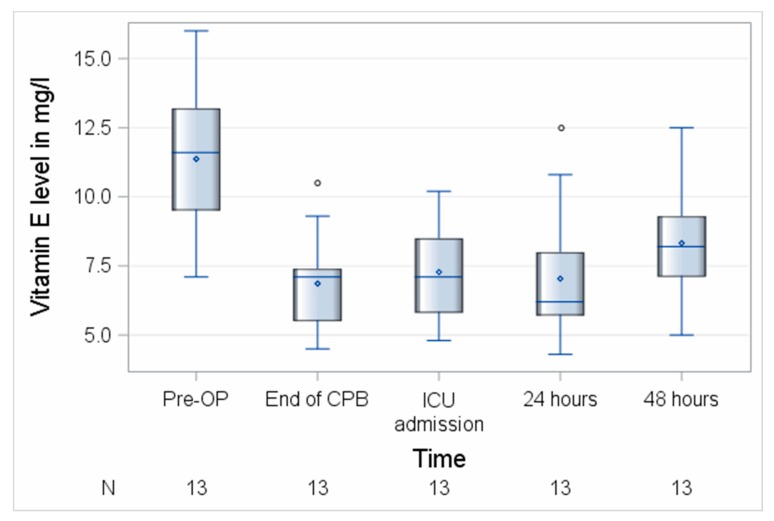
Perioperative course of vitamin E. CPB, cardiopulmonary bypass; ICU, intensive care unit; N, number of patients. Circles represent outliers.

Both vitamins decreased significantly during surgery with a median loss of 2.6 (0.7–5.3) mg/L, *p* = 0.0002 (vitamin C) and 4.3 (3.4–5.5) mg/L, *p* = 0.0002 (vitamin E) corresponding to 66% (vitamin C) and 63% (vitamin E) of the respective baseline levels. Vitamin C levels remained significantly reduced until 48 h after surgery to (36%, *p* < 0.0001), while blood levels of vitamin E remained reduced for 24 h and then recovered at 48 h (72%, *p* = 0.0005) of their baseline levels as shown in Figure 2. Fifty-six percent of patients (19/34) had a preoperatively suboptimal vitamin C status and no patient was vitamin E deficient before surgery. In total, 65.6% (21/32) of the patients had a perioperative loss of vitamin C of ≥50%.

### 3.3. Influence of Probe Handling on the Absolute Values of Vitamin C

**Figure 3:**
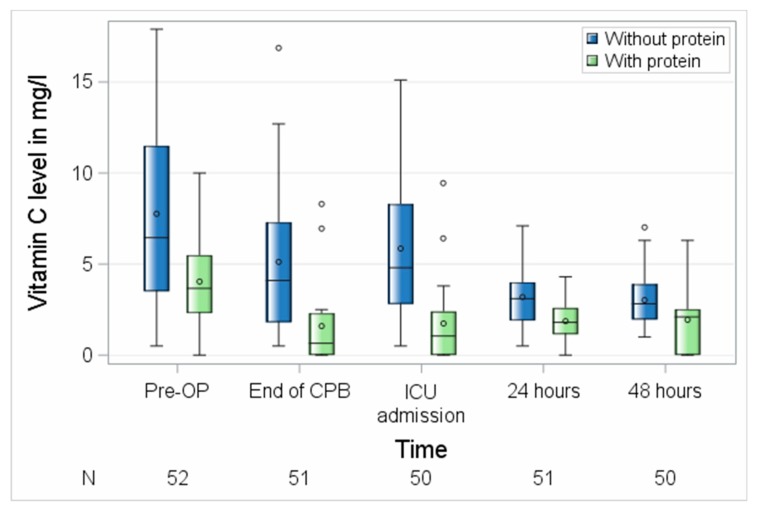
Perioperative course of vitamin C with different probe handling. CPB, cardiopulmonary bypass; ICU, intensive care unit; N, number of patients. Circles represent outliers.

Next, we investigated the influence of probe handling on the measured vitamin C levels in the blood. As shown in Figure 3 and Table 2, the absolute and relative values of vitamin C strongly depended on the performed procedures. While in the probes of the first 22 patients the mean absolute values were very low (3.1 (1.5–4.3) mg/L before surgery and 1.9 (0.0–2.5) ± 1.6 mg/L 48 h after surgery), the absolute values of vitamin C in the protein-denaturized probes were significantly higher (6.5 (3.5–11.5) mg/L before surgery (*p* = 0.0008) and 2.8 (2.0–3.9) mg/L 48 h after surgery (*p* = 0.0030)).

### 3.4. The Association of Vitamin C with Inflammation

**Figure 4:**
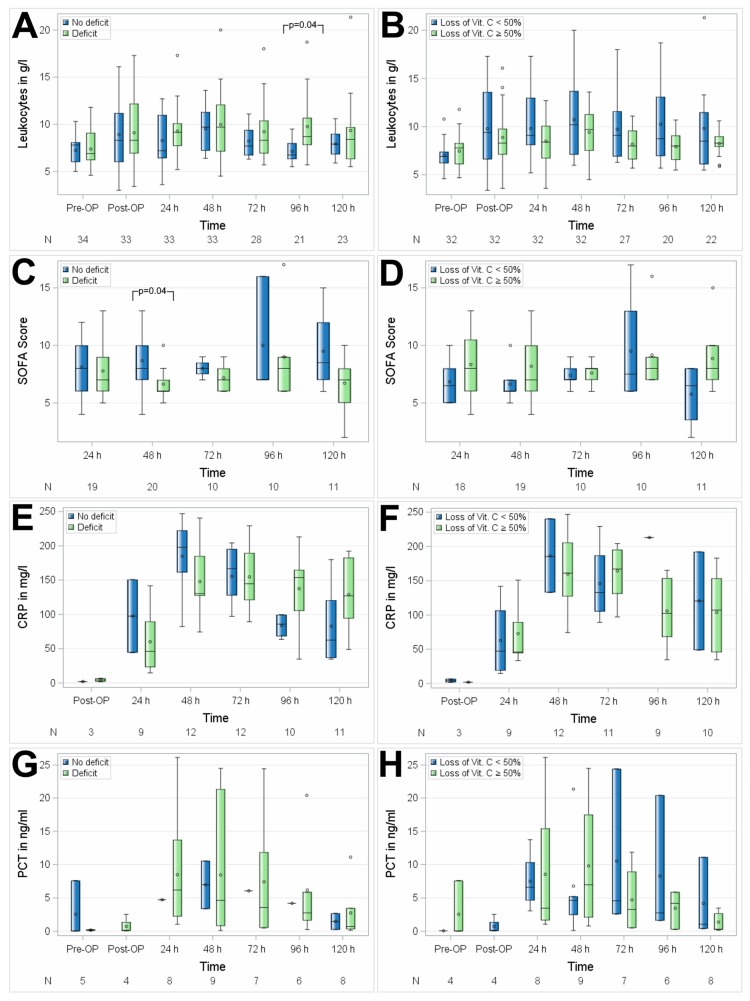
Association of perioperative vitamin C levels with leukocytes (**A**,**B**), SOFA Score (**C**,**D**), CRP (**E**,**F**), and PCT (**G**,**H**). N, number of patients; (**A**,**C**,**E**,**G**) patients with preoperative vitamin C deficit; (**B**,**D**,**F**,**H**) patients with intraoperative loss of vitamin C ≥ 50%. Circles represent outliers.

No significant differences between the groups with suboptimal preoperative vitamin C status versus preoperative normal plasma serum levels of vitamin C were observed regarding postoperative inflammatory response, as assessed by mean postoperative white WBC levels (*p* = 0.6773), PCT levels (*p* = 0.4902), and SOFA Score (*p* = 0.1033). The CRP was measured in both groups of patients, when the attending medical staff indicated their measurement (*n* = 3 after surgery, *n* = 9 the first postoperative day, *n* = 12 at the second postoperative day, *n* = 12 at the third postoperative day, *n* = 10 at the fourth postoperative day, and *n* = 11 at the fifth postoperative day) levels were higher in patients with suboptimal preoperative vitamin C status at 96 and 120 h after surgery. PCT measurement as indicated by the attending medical staff was performed on 5 patients before surgery, 4 patients after surgery, 8 patients the first postoperative day, 9 patients at the second postoperative day, 7 patients at the third postoperative day, 6 patients at the fourth postoperative day, and 8 patients at the fifth postoperative day. Patients with a perioperative loss of vitamin C ≥ 50% compared to patients with perioperative loss < 50% did not show a difference with respect to the postoperative inflammatory response as assessed by mean postoperative WBC (*p* = 0.6625), CRP (*p* = 0.1318) and PCT levels (*p* = 0.5804), and SOFA Score (*p* = 0.1699), whereas the latter showed higher mean values of SOFA Score in patients with significant intraoperative vitamin C loss during the postoperative course, which however did not reach statistical significance (Figure 4A,B).

**Figure 5:**
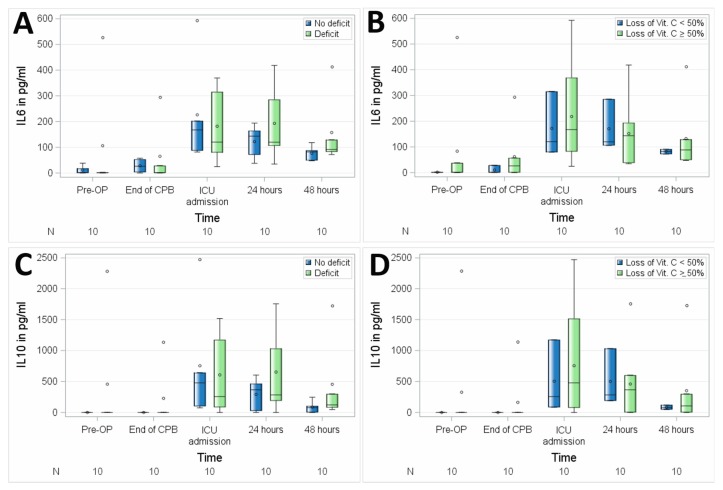
Association of perioperative vitamin C levels with interleukin 6 (IL6) (**A**,**B**) and interleukin 10 (IL10) (**C**,**D**). N, number of patients; (**A**,**C**), patients with preoperative vitamin C deficit; (**B**,**D**), patients with intraoperative loss of vitamin C ≥ 50%. Circles represent outliers.

No significant differences between the groups with suboptimal preoperative vitamin C status versus preoperative normal plasma serum levels of vitamin C were observed regarding the perioperative course of interleukins, as assessed in 10 patients by mean IL6 (*p* = 0.7514) and IL10 (*p* = 0.7261) as shown in Figure 5 and Table 3.

### 3.5. The Association of Vitamin C with Oxidative Stress

**Figure 6:**
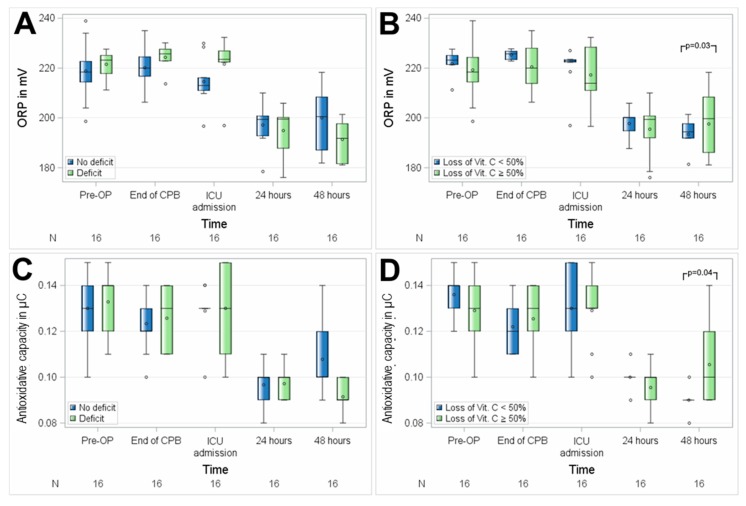
Association of perioperative vitamin C levels with oxidative stress, assessed as oxidation–reduction potential (ORP) (**A**,**B**) and anti-oxidative capacity (**C**,**D**). N, number of patients; (**A**,**C**), patients with preoperative vitamin C deficit; (**B**,**D**), patients with intraoperative loss of vitamin C ≥ 50%. Circles represent outliers.

No significant differences between the groups with suboptimal preoperative vitamin C status and preoperative normal plasma serum levels of vitamin C were observed regarding perioperative oxidative stress, as assessed by mean ORP (*p* = 0.6720) and AC (*p* = 0.4859) in 16 patients. Patients with a suboptimal preoperative vitamin C status showed visually higher ORP levels at the end of CPB and at ICU admission, which did not reach statistical significance in this small group of patients (Figure 6). No differences between ORP levels and anti-oxidative capacity were measured between patients with intraoperative vitamin C loss ≥ 50% and others at any of the perioperative time points.

### 3.6. The Association of Vitamin C with Organ Dysfunction

**Figure 7:**
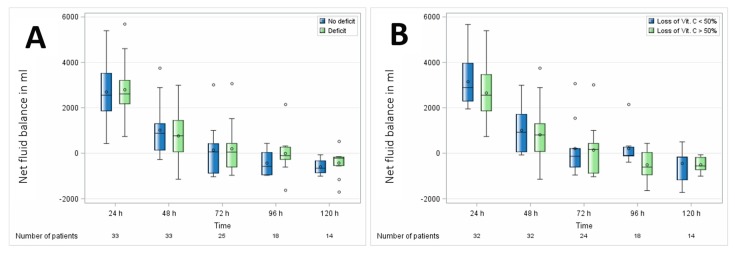
Association of perioperative vitamin C levels with fluid balance. (**A**) Patients with preoperative vitamin C deficit and (**B**) patients with intraoperative loss of vitamin C ≥ 50%. N, number of patients. Circles represent outliers.

Overall, no significant association of vitamin C levels with organ dysfunction was observed in either of the comparisons, as displayed in Table 4. Vitamin C had no association with different postoperative hemodynamics, reflected by fluid and vasopressor demand as displayed in Figure 7.

### 3.7. The Association of Vitamin C with Length-of-Stay and Mortality

In this explorative study, vitamin C plasma levels were not significantly associated with ICU or hospital length-of-stay or with hospital or 30-day mortality, as shown in Table 5. Shorter ICU length-of-stays were observed in these patients, who had higher preoperative vitamin C serum levels and in patients with smaller perioperative decreases of vitamin C.

## 4. Discussion

In this prospective observational study, perioperative levels of vitamins C and E were described and the association of perioperative vitamin C levels with clinical outcomes was evaluated in an explorative approach. We observed significant decreases in both vitamins during and after surgery. While vitamin E levels began to recover 48 h after surgery, vitamin C levels remained low. Probe handling had a strong influence on the measured levels of vitamin C. In protein-denaturized probes, significantly higher vitamin C levels were measured. Clinical evaluation showed no statistically significant association of vitamin C levels with inflammation, oxidative stress, organ dysfunction, length-of-stay, and mortality. However, the small number of patients, and measurements of the respective markers, as well as the elective nature of the surgeries do not allow for generalization of the results of this small explorative study.

Our results are in line with the findings of Lassnigg et al., who also observed sharp decreases of both vitamin C and E after reperfusion in 24 patients [6]. Ballmer et al. as well as Rodemeister et al. also observed a large decrease of 71% of vitamin C in 18 and 29 patients undergoing cardiac surgery, which did not recover within 24 h after surgery [28] or even 6 days after surgery [29]. In the study by Ballmer et al., one-third of their patients also showed preoperative low plasma concentrations of <23 µmol/L, putting emphasis on preoperative suboptimal vitamin status, which might be easily prevented [28]. Ballmer et al. observed a 40%-decrease of absolute vitamin E values as well, which was not significant when corrected with lipid-standardized plasma. The decrease of vitamin C and E serum levels after CPB may indicate either a binding to the CPB, distribution to different compartments, or a consumption of antioxidants during reperfusion of previously ischemic tissues during impediment of aortic blood flow through a cross clamp.

Although remaining speculative, the spontaneous increase of vitamin E plasma levels at 48 h can be interpreted as vitamin E restoration by physiologic shifts between the compartments, as well as by vitamin E recycling via other molecules, such as ubiquinol and vitamin C. Therefore, it is not surprising that vitamin E levels recovered, while vitamin C levels remained significantly decreased, possibly leading to a suboptimal anti-oxidative and anti-inflammatory response mechanism in these patients after surgery. In fact, these results raise the question, if significantly reduced vitamin C levels increase the concerning patient’s vulnerability for infectious complications and the development of organ dysfunctions during the prolonged ICU-stay. Yet, this study was not designed for answering this clinically relevant question.

Present findings further question if the perioperative supplementation of vitamins C and E will prevent the decrease of their plasma levels and will increase antioxidant, anti-inflammatory, and immunological defense mechanisms [11,18,20]. In this context, the clinical relevance of the plasma levels of these two antioxidant nutrients need to be evaluated more carefully—not only during the acute perioperative phase, but as well as during the prolonged ICU stay in a larger cohort of higher-risk patients. Importantly, the postoperatively measured higher mean SOFA score in patients with severe vitamin C deficiency and the fact that half the patients were vitamin C deficient prior to surgery raise the question, if a preoperative optimization of these micronutrients would be beneficial for this group of patients undergoing elective and scheduled cardiac surgery.

However, micronutrient supplementation is currently recommended only in patients with proven deficiency, not as a routine for all patients undergoing surgery [30,31,32]. On the other hand, the measurement of vitamins C and E is expensive, is not available in most hospitals, and probe handling involved in their measurements was observed to have a strong impact on the values, and the measurement of the UV-, thermo-, and pH-sensitive vitamin C will be challenging in clinical routine, which remains largely unrecognized in ongoing clinical studies. Additionally, in the absence of fast bedside analyses, the needed laboratory values will not be obtained fast enough to perioperatively supplement those patients with proven deficits.

Therefore, a validated marker to rapidly assess overall redox-stress, as well as the effects of a possible antioxidant therapy is urgently required. One possible bedside-tool might be the oxidation–reduction potential, which is known to assess the overall balance of oxidants and reductants in the blood and which is associated with severity of disease and patient outcomes in patients with polytrauma and traumatic brain injury [33,34], as well as in pediatric patients after cardiac surgery [35]. In the present study, our explorative analysis suggests higher levels of oxidative stress in patients with preoperative suboptimal vitamin C status directly after the termination of CBP and ICU admission, reflecting time points with maximal release of reactive oxygen and nitrogen species at the end of myocardial ischemia/reperfusion and termination of the surgical trauma. The higher levels of ORP measured in patients with preoperative suboptimal vitamin C status thus may indicate less efficient anti-oxidative defense mechanisms, which may lead to the development of major organ dysfunctions such as acute kidney injury in these patients.

Our results regarding the oxidative stress are in line with the recently published study by Rozemeijer et al., who also observed an increase of oxidative stress in ICU-patients undergoing major surgery/sepsis or trauma, as well as in patients after cardiac arrest to similar levels we observed in patients undergoing cardiac surgery. Additionally, Rozemeijer et al. observed a strong negative association of ORP and strong positive association between the anti-oxidative capacity with plasma vitamin C concentrations (R^2^ > 0.8 for ORP and R^2^ = 0.842 for anti-oxidative capacity) and demonstrated a strong concordance between changes in plasma vitamin C concentration and changes in severity of oxidative stress, reflecting that a high dose of vitamin C diminished the oxidative stress in their critically ill patients [36].

Regarding the clinical outcome of the patients, no significant association with vitamin C levels were observed in this observational explorative study, beside the non-significant postoperative higher SOFA score in patients with preoperative suboptimal vitamin C status. In fact, we acknowledge that it is challenging to detect clinically meaningful and measurable associations in low-risk patients and small cohort, who are frequently discharged early after 1–2 days and reflected by the rare occurrence of organ dysfunction, short ICU and hospital length-of-stays, and 30-day mortality [37,38]. These elective patients undergoing mostly low-risk open-heart surgery are expected to rapidly recover after surgery and often stay in the ICU for one night only, irrespective of any additional treatment. These patients might also be normally nourished before and after surgery, allowing for adequate supply of macro- and micronutrients. In contrast, patients undergoing more complex heart surgeries, for example, combined cardiopulmonary artery bypass graft and valve surgery or patients receiving a ventricular assist device have an increased risk for longer surgeries and CPB-times, as well as prolonged recoveries. These patients, as well as patients with preoperative malnutrition might be more susceptible for malnutrition and perioperative vitamin deficits, which deserves further investigation.

The ICU length-of-stay was 24 h in patients with higher vitamin C levels than in the comparator-groups (48 h)—both in patients with higher preoperative levels, as well as in patients with smaller intraoperative vitamin C loss. Although these results did not reach statistical significance, this might be of clinical relevance, especially for patients with prolonged ICU-stay [39], and needs to be investigated in future studies. Regarding this topic, several meta-analyses were published, discussing the potential benefits of vitamin C administration in patients undergoing cardiac surgery [40,41,42,43,44,45,46], as well as in critically ill patients [47,48,49,50,51,52] and many observed associations with shorter hospital and ICU length-of-stays, decreased duration of mechanical ventilation and vasopressor support, and lower mortality.

Our study has several limitations. The sample size was too small to allow for any conclusions regarding the association of the vitamin levels with the clinical outcome. Considering the measurement of IL6, IL10, and ORP, we must acknowledge that the present data should be interpreted cautiously within the limitations of an explorative analysis, which should be considered hypothesis-generating due to the low number of observations. Secondly, we included all patients consecutively without risk-stratification, therefore, recording low frequencies of adverse events, such as organ dysfunction or mortality in elective patients. Thirdly, we observed a high inter-individual variability of vitamin C, leading to the need of studies with greater patient numbers to draw meaningful conclusions. Fourthly, intraoperative treatments, such as the administration of blood products and colloids may have influenced the patient´s native vitamin C and E levels. Furthermore, as our laboratory values other than vitamins, oxidative status, and interleukins were taken as part of clinical routine, and all collected blood samples were used for these purposes, it was not possible for us to correct the vitamin E levels with blood lipids, or to perform additional measurements, for example, for other inflammatory markers. Yet, present finding of a significant decrease of these key micronutrients demonstrates the need for a more carefully clinical evaluation, especially in the post-acute phase of cardiac surgery patients.

## 5. Conclusions

In conclusion, while the vitamin C and E plasma levels decrease dramatically during elective cardiac surgery with cardiopulmonary bypass and remain low in the first two postoperative days, their influence on clinical outcome remains unclear in this small observational study. The clinical relevance of vitamin C and E deficiency in patients undergoing cardiac surgery with cardiopulmonary bypass requires further investigation.

## Figures and Tables

**Table 1:** Baseline characteristics of patients included in clinical analysis. BMI, body mass index; CPB, cardiopulmonary bypass; EuroScore, preoperative European System for Cardiac Operative Risk Evaluation Score; EF%, preoperative ejection fraction; CABG, coronary artery bypass graft; * clinical signs of heart failure as assessed by the New York Heart Association Score Class II–IV; N, number of patients, values presented as median [interquartile range] or amount (%).

	Preoperative Vitamin C Status				Perioperative Loss of Vitamin C			
Baseline Characteristic	*N*	<9 mg/L (*N* = 19)	≥9 mg/L (*N* = 15)	*p*-value	*N*	≥50% (*N* = 21)	<50% (*N* = 11)	*p*-value
Age in Years	34	69 (62–75)	72 (66–78)	0.2816	32	72 (62–77)	69 (64–71)	0.2106
Weight in kg	34	75 (68–90)	78 (68–96)	0.7415	32	78 (68–95)	80 (70–98)	1.0000
Height in m	34	170 (163–177)	170 (165–182)	0.6144	32	169 (165–176)	172 (163–180)	0.6334
BMI in kg/m2	34	26.9 (24–30.8)	27.2 (23.5–29.3)	0.9862	32	27.2 (23.5–29.7)	26.1 (22.3–34.3)	0.6198
Duration of Surgery	33	236.5 (181–278)	198 (185–258)	0.3855		211 (196–282)	234 (163–277)	0.6915
Duration of Aortic Cross Clamp	33	67 (54–95)	62 (47–68)	0.1866	32	64 (50–75)	62 (47–95)	0.8117
Duration of CPB	33	123.5 (86–171)	102 (83–116)	0.1930	32	104 (85–123)	124 (79–171)	0.7062
Sex (Female)	34	8 (42%)	5 (33%)	0.7282	32	7 (33%)	5 (45%)	0.7026
Heart Failure *	34	4 (21%)	1 (7%)	0.3547	32	2 (10%)	2 (18%)	0.5932
EuroScore	7	7.1 (4.5–9.6)	6.6 (6.5–18.4)	0.5959	6	7.7 (5.8–13.7)	7 (3.7–10.3)	0.8170
EF%	21	50.5 (38.3–60.5)	56 (50–61)	0.2697	19	56.5 (49.5–61.5)	56 (39–64)	0.4714
Type of Surgery	34			0.7886	32			0.2445
Valve		6 (31.6%)	5 (33.3%)			7 (33.3%)	2 (18.2%)	
CABG		10 (52.6%)	9 (60.0%)			13 (61.9%)	6 (54.6%)	
Combined/Other		3 (15.8%)	1 (6.7%)			1 (4.8%)	3 (27.3%)	

**Table 2:** Description of perioperative values of vitamin C with different probe treatments. Vit, vitamin; N, number of patients; Min, minimum; Pctl, percentile; Max, maximum; N, number of patients.

Sample	Variable	N	20th Pctl	Lower Quartile	40th Pctl	Median	60th Pctl	Upper Quartile	80th Pctl
With protein	Pre-OP Vit C in mg/L	17	2.3	2.58	3.56	3.73	4.1	5.49	6.4
	Loss of Vit C in %	16	13	21	39	50	69	85	100
	Individual slope of Vit C	17	−0.85	−0.78	−0.55	−0.4	−0.04	−0.02	−0.02
Without protein	Pre-OP Vit C in mg/L	34	3	3.5	5.7	6.45	9.4	11.5	12.1
	Loss of Vit C in %	32	35	39	57	64	66	69	71
	Individual slope of Vit C	34	−1.96	−1.88	−1.5	−1.1	−0.67	−0.4	−0.23

**Table 3:** Correlation between vitamin C and interleukin 6 (IL6) and interleukin 10 (IL10).

Spearman rho	Pre-OP	Post-OP	ICU Admission	24 h	48 h	Mean	*p*-Value
IL6	−0.018	0.024	0.055	−0.152	−0.529	−0.11515	−0.115
IL10	−0.2381	−0.52382	−0.00606	−0.19453	−0.54269	−0.12727	−0.127

**Table 4:** Association of perioperative vitamin C levels with organ dysfunction. ARDS, acute respiratory distress syndrome; AF, new postoperative atrial fibrillation; N, number of patients. Data presented as median (interquartile range) or amount (%).

	Preoperative Vitamin C Status				Perioperative Loss of Vitamin C			
Outcome	N	<9 mg/L (*N* = 19)	≥9 mg/L (*N* = 15)	*p*-Value	*N*	≥50% (*N* = 21)	< 50% (*N* = 11)	*p*-Value
Duration of Mech. Ventilation in Hours	30	10.1 (6.4–17.6)	10.6 (8.5–12.2)	0.9337	29	10.8 (7.3–13.8)	9.8 (6.5–16)	0.9624
Cumulative Post-OP Norepinephrine in µ/kg	34	1 (0.6–3.0)	0.8 (0.3–1.9)	0.2981	32	1.0 (0.6–1.9)	1.1 (0.6–6.1)	0.4509
Cumulative Post-OP Adrenaline in µ/kg	3	9.1 (4.2–14.1)	14.1 (14.1–14.1)	0.5403	3	14.1 (14.1–14.1)	9.1 (4.2–14.1)	0.5403
Net Fluid Balance 72 h Post-OP in mL	25	3119 (1930–3959)	3963 (2191–4750)	0.722	24	3959 (2191–4750)	3101 (2782–3376)	0.7656
Delir	33	4 (22%)	1 (7%)	0.3457	32	2 (10%)	3 (27%)	0.3098
ARDS	33	0 (0%)	0 (0%)		32	0 (0%)	0 (0%)	
Stroke	33	1(6%)	0 (0%)	1.0000	32	1 (5%)	0 (0%)	1.0000
AF	33	5 (28%)	3 (20%)	0.6992	32	5 (24%)	3 (27%)	1.0000
Acute Kidney Injury	33	3 (17%)	2 (13%)	1.0000	32	2 (10%)	3 (27%)	0.3098
Acute Dialysis	33	1 (6%)	1 (7%)	1.0000	32	1 (5%)	1 (9%)	1.0000
Infection	33	4 (22%)	1 (7%)	0.3457	32	2 (10%)	3 (27%)	0.3098

**Table 5:** Association of perioperative vitamin C levels with length-of-stay and mortality. N, number of patients, presented as median (interquartile range) or amount (%).

	Preoperative Vitamin C Status				Perioperative Loss of Vitamin C			
Outcome	*N*	<9 mg/L (*N* = 19)	≥9 mg/L (*N* = 15)	*p*-Value	*N*	≥50% (*N* = 21)	< 50% (*N* = 11)	*p*-Value
**ICU Stay in Hours**	32	135 (60–149)	72 (28–147)	0.199	31	75 (45–149)	129 (74–141)	0.3524
**Hospital Stay in Days**	32	9 (7–13)	8 (7–13)	0.5168	31	8 (7–13)	10.5 (9–13)	0.2943
**30-Day Mortality**	34	2 (11%)	0 (0%)	0.492	31	0 (0%)	1 (9%)	0.3437
